# Growth Factor-Reinforced ECM Fabricated from Chemically Hypoxic MSC Sheet with Improved In Vivo Wound Repair Activity

**DOI:** 10.1155/2017/2578017

**Published:** 2017-09-05

**Authors:** Hui-Cong Du, Lin Jiang, Wen-Xin Geng, Jing Li, Rui Zhang, Jin-Ge Dang, Mao-Guo Shu, Li-Wen Li

**Affiliations:** ^1^Key Laboratory of Resource Biology and Biotechnology in Western China, Northwest University, Ministry of Education, Xi'an, Shaanxi 710069, China; ^2^Department of Plastic and Burn Surgery, Tangdu Hospital, Forth Military Medical University, Xi'an, Shaanxi 710038, China; ^3^Department of Plastic Surgery, The First Affiliated Hospital of Xi'an Jiaotong University, Xi'an, Shaanxi 710061, China

## Abstract

MSC treatment can promote cutaneous wound repair through multiple mechanisms, and paracrine mediators secreted by MSC are responsible for most of its therapeutic benefits. Recently, MSC sheet composed of live MSCs and their secreted ECMs was reported to promote wound healing; however, whether its ECM alone could accelerate wound closure remained unknown. In this study, Nc-ECM and Cc-ECM were prepared from nonconditioned and CoCl_2_-conditioned MSC sheets, respectively, and their wound healing properties were evaluated in a mouse model of full-thickness skin defect. Our results showed that Nc-ECM can significantly promote wound repair through early adipocyte recruitment, rapid reepithelialization, enhanced granulation tissue growth, and augmented angiogenesis. Moreover, conditioning of MSC sheet with CoCl_2_ dramatically enriched its ECM with collagen I, collagen III, TGF-*β*1, VEGF, and bFGF via activation of HIF-1*α* and hence remarkably improved its ECM's in vivo wound healing potency. All the Cc-ECM-treated wounds completely healed on day 7, while Nc-ECM-treated wounds healed about 85.0% ± 8.6%, and no-treatment wounds only healed 69.8% ± 9.6% (*p* < 0.05). Therefore, we believe that such growth factor-reinforced ECM fabricated from chemically hypoxic MSC sheet has the potential for clinical translation and will lead to a MSC-derived, cost-effective, bankable biomaterial for wound management.

## 1. Introduction

Wound healing is one of the most complex and dynamic biological processes that occurs in human lifespan [1, 2]. Improper or impaired wound healing not only leads to significant morbidity and mortality but also places immense burden on healthcare systems worldwide. Because of the great importance and demand for wound management products, skin substitute biomaterials have attracted more and more intensions in recent years. And a large variety of biomaterials capable of promoting wound healing have been derived from allogeneic or xenogeneic resources, chemical or recombinant materials, or a combination of both [3, 4]. Among these materials, natural resource-derived extracellular matrices (ECMs) are of special interest since such ECMs can provide structural support and scaffolding as well as biochemical signals for tissues and cells simultaneously [5]. Acellular dermal matrix (ADM), the natural ECM fabricated from decellularized dermal tissue, has long been used to replace lost skin tissues in clinical settings [6, 7]. However, ADM contains only low doses of native growth factors which can hardly be increased due to ethical concerns [8]. Therefore, natural or artificial ECM alternatives enriched in growth factors are worthwhile pursuing [5].

Cell sheet technology is a novel strategy to fabricate scaffold-free 3D tissues and shows extraordinary potency in tissue engineering and regenerative medicine [9, 10]. Recently, cell sheets derived from mesenchymal stem cells (MSCs) isolated from adipose tissue were reported to accelerate cutaneous wound healing by promoting reepithelialization and revascularization [11–13]. However, their translating from bench-top to clinic is limited by some drawbacks, such as the risk of aberrant immune responses and cost implications for maintaining cell viability in stringent storage and transport conditions. Accumulating evidences demonstrated that MSCs participate in tissue repair and regeneration mainly through their secretome instead of direct differentiation into local cell types as once thought [14, 15]. Among MSC's secretome, ECM is mainly composed of collagens and acts as a reservoir for growth factors which can be rapidly mobilized to stimulate wound repair [16, 17]. Therefore, ECM seems to be a better choice in comparison to its original cell sheet because it is cost-effective, readily available, and devoid of cellular antigens. Moreover, it might be feasible to manipulate the reservoir of growth factors trapped in the ECM and hence its wound healing potency.

Hypoxia-inducible factor-1 (HIF-1) is a well-known transcription factor that mediates the cellular adaptive responses to hypoxia and plays critical roles in the process of wound repair [18, 19]. It not only regulates the expression of several ECM-bound growth factors, including VEGF, bFGF, and TGF-*β*1 [20], but also participates in regulation of collagen deposition, fiber alignment, and ECM stiffening [21]. In addition to low oxygen tension exposure, cobalt chloride (CoCl_2_), the widely used chemical hypoxia inducer, is well documented to activate HIF-1 by increasing the stability of its oxygen labile *α* subunit [22]. Therefore, we proposed that CoCl_2_ conditioning of MSC sheet could enrich its ECM with growth factors via a HIF-1*α*-dependent pathway and thereby improved its wound healing properties.

In this study, the effects of CoCl_2_ conditioning on the expression of collagens and growth factors in MSC sheets and their derived ECMs were investigated. And the in vivo wound healing efficacy of the enriched ECMs derived from chemically hypoxic MSC sheets was evaluated in a mouse model of full-thickness skin defect.

## 2. Materials and Methods

### 2.1. Isolation of MSCs

Two-week-old New Zealand rabbits (*n* = 4) were obtained from Animal Center of Fourth Military Medical University. Rabbit bone marrow MSCs were isolated in accordance with IACUC approval of Northwest University and cultured as reported previously with minor modifications [23]. MSCs were cultured in DMEM/F12 medium (Corning, USA) with 10% fetal bovine serum (MP Biomedicals, USA), with 0.272 g/L L-glutamine (Sigma, USA), 100 *μ*g/mL penicillin and 100 *μ*g/mL streptomycin at 37°C, and 5% CO_2_. Cells at passage 2 were used for all experiments.

### 2.2. Formation of Cell Sheets and Chemical Hypoxia Conditioning

Primary MSCs were seeded at 20,000 cells per cm^2^ in complete medium supplemented with 100 *μ*g/mL vitamin C (Sigma, USA). Half of the medium was changed daily. Seven days later, chemical hypoxia conditioning was performed by adding CoCl_2_ to the culture medium to final concentration of 100 *μ*M. After another 7-day culture, cell sheets were intactly harvested from the Petri dishes and their derived ECMs were achieved by a standard decellularization procedure [24].

### 2.3. Fabrication of ECMs from Cell Sheets

Decellularization was conducted in warm double distilled water supplemented with 20 mM ammonium hydroxide (Sigma, USA) for 10 min with shaking on a rotary shaker. After being washed with cold PBS for three times, the resulting cell-free ECMs were treated with DNase I (Sigma, USA) for 30 min at 37°C to eliminate DNA residues. Then DNA-free ECMs were washed with PBS for five times and stored at −20°C for 4 weeks before use.

### 2.4. Western Blot Analysis

All the MSC sheets and their derived ECMs were cut into pieces and then were ground in a glass homogenizer on ice in cold RIPA buffer (with phenylmethylsulfonyl fluoride). After centrifugation for 15 min at 4°C, 14,000 ×g, the supernatants were transferred to new tubes. Protein concentrations were evaluated by BCA protein assay kit (TaKaRa, Japan). Equal amounts of total protein of different samples were loaded for western blot analysis using standard procedures. HIF-1*α* antibody, collagen I *α*1 antibody, and collagen III *α*1 antibody were purchased from NOVUS Biologicals (USA), TGF-*β*1 antibody was purchased from Abcam (USA), and VEGF antibody, bFGF antibody, *β*-actin antibody, mouse anti-rabbit IgG-HRP, and goat anti-mouse IgG-HRP were purchased from Santa Cruz Biotech (USA). The protein levels were normalized by using *β*-actin as an internal control.

### 2.5. Mouse Models of Full-Thickness Skin Defect Wounds

Female Balb/C mice (8~10 weeks old, *n* = 48) were obtained from Animal Center of Fourth Military Medical University. All in vivo experiments were approved by IACUC of Northwest University. The mice were randomly divided into 3 groups: the control group received no treatment and experimental groups were treated with ECMs fabricated from nonconditioned MSC sheets (Nc-ECMs) or ECMs from CoCl_2_-conditioned MSC sheets (Cc-ECMs). The in vivo wound healing experiment was conducted in a mouse model of full-thickness skin defect [25]. Briefly, two 6 mm diameter full-thickness wounds were created on the back of each mouse. Silicone rubber ring with inner diameter of 6 mm was adhered to the skin around the wound to prevent wound from shrinkage and to ensure wound healed through granulation tissue formation and reepithelialization, a process similar to that occurring in humans [25]. And ECMs of 7-mm diameter were transplanted onto the wound beds immediately after wounding. Then all the wounds were covered with Vaseline gauze to prevent ECM from drying and dressed with self-adhering elastic bandages. The photographs of the wounds were taken on days 0, 3, 5, and 7 using Canon Digital Camera (EOS 700D with Macro Lens EF-S 60 mm f/2.8 USM, Canon, Inc., Tokyo, Japan) and analyzed for healing progress using Image J software.

### 2.6. Histological Analysis

All samples were fixed overnight in 4% paraformaldehyde at 4°C. After alcoholic dehydration and paraffin embedded, the samples were sectioned at 5 *μ*m using a Leica RM2235 rotary microtome. Hematoxylin and eosin (H&E) staining, Masson's trichrome (MTC) staining, and picrosirius red (PSR) staining were performed and the sections were observed under a Leica DMI 6000 B automated fluorescence microscope or a Leica M205 FA stereomicroscope.

### 2.7. Statistical Analysis

All data were given as mean ± s.d. Statistical evaluation was performed using one-way analysis of variance (ANOVA), followed by Dunnett's *t*-test for experimental groups and control group comparisons and Bonferroni's test for experimental groups comparison. The results of western blot were analyzed by independent-sample *T* test.

## 3. Results

### 3.1. Characteristics of MSC Sheets and Their Derived ECMs

**Figure 1:**
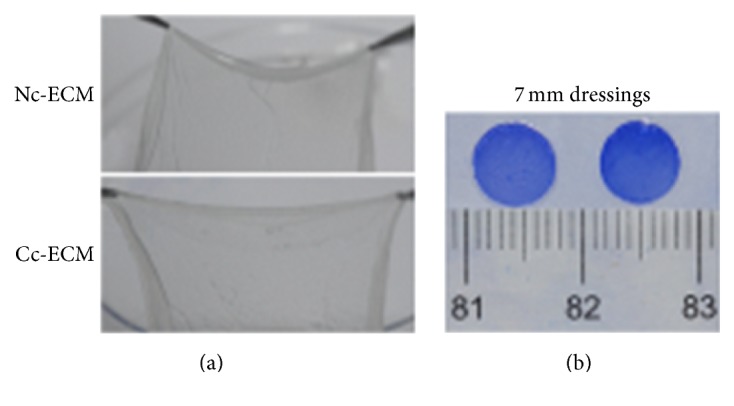
Morphology of MSC sheets derived ECMs. Representative macroscopic images of Nc-ECMs and Cc-ECMs (a). The 7 mm diameter ECM dressings were stained with coomassie blue dye for 5 minutes (b).

**Figure 2:**
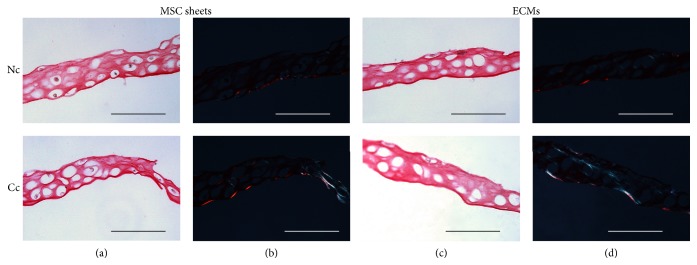
PSR staining of MSC sheets and their derived ECMs. Imaging of MSC sheets and ECMs under light microscopy (a, c). Imaging of birefringent collagen through crossed polarization light microscopy. Large orange-red fibers were collagen I and thin blue-green filamentous fibers were collagen III (b, d). Scale bars represent 100 *μ*m.

All the acellular ECMs were semitransparent and no macroscopic differences were identified between different groups. The ECMs could be easily handled with forceps and cut into round shape, indicating that they were mechanically stable enough for in vitro manipulation (Figure 1). PSR staining showed that all cell sheets were composed of 2-3 layers of MSCs embedded in large amounts of ECMs (Figure 2(a)). The red-stained collagens observed in bright-field microscopy and the green-stained collagen III in polarization microscopy were more significant in both CoCl_2_-conditioned MSC sheet and its derived Cc-ECM than those in nonconditioned controls (Figures 2(b) and 2(d)). Moreover, decellularization removed most of MSCs and resulted in ECMs with highly porous microstructures (Figure 2(c)).

### 3.2. CoCl_2_ Activates HIF-1*α* in MSC Sheets

**Figure 3:**
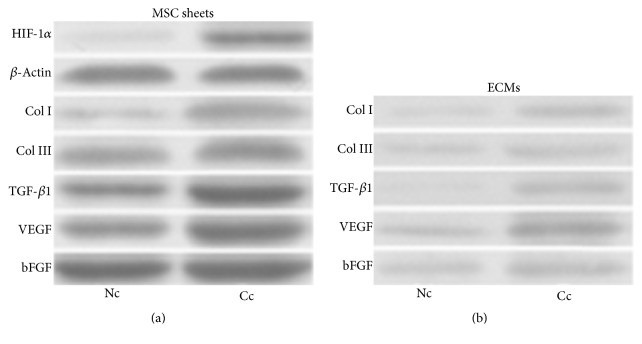
Western blot analysis of HIF-1*α*, collagens, and growth factors in MSC sheets and their derived ECMs. The proteins expressed in MSC sheets (a). The proteins expressed in their derived ECMs. The equal amounts of total protein in two groups were loaded for western blot analysis (b).

**Figure 4:**
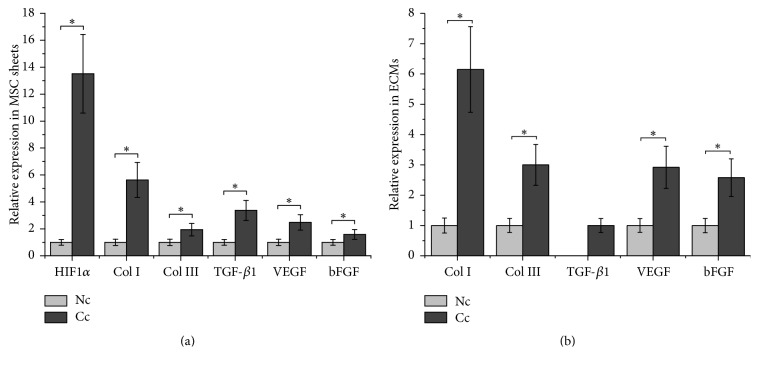
Effects of CoCl_2_ on protein expression. The relative protein levels of HIF-1*α*, collagens, and growth factors in MSC sheets (a). The relative protein levels of collagens and growth factors in ECMs (b). ^*∗*^*p* < 0.001, *n* = 8.

HIF-1*α* is subject to continuous proteasomal degradation under normoxic conditions and its protein level elevates quickly in response to hypoxic stress. In addition to culture cells under hypoxia conditions, CoCl_2_ has been widely used to induce chemical hypoxia in many other cell types [26–30]. In this study, CoCl_2_ exposure significantly increased the protein level of HIF-1*α* in MSC sheets (Figure 3(a)), and a 13.5-fold increase was detected in our study, indicating that chemical hypoxia was successfully induced in MSC sheets (Figure 4(a)).

### 3.3. CoCl_2_ Enriches Collagens and Growth Factors in ECMs

Many collagen-based biomaterials have been developed as wound biodressings and showed positive outcomes in preclinical and clinical wound management [31]. And collagen I and collagen III are known to be involved in the process of wound healing [32]. We showed that CoCl_2_ conditioning induced a 5.6-fold increase in collagen I expression and a 1.9-fold increase in collagen III relative to the nonconditioned MSC sheet (Figures 3(a) and 4(a)). And after decellularization, the retained amounts of collagen I and collagen III in Cc-ECM were approximately 6.1-fold and 3.0-fold of that in Nc-ECM, respectively (Figures 3(b) and 4(b)). These results indicated that CoCl_2_ not only upregulated the expression of collagen I and collagen III in MSC sheets but also enhanced their preservation in the resulting acellular ECMs.

Since TGF-*β*1 plays important roles in reepithelialization as well as collagen deposition through regulating fibroblast and myofibroblast activities [16, 33], we also assessed the effect of chemical hypoxia conditioning on its expression. The results demonstrated that CoCl_2_ induced a 3.4-fold increase in TGF-*β*1 level in MSC sheet (Figures 3(a) and 4(a)). However, most TGF-*β*1 was lost during decellularization and trace amount of TGF-*β*1 was detected only in the Cc-ECM (Figures 3(b) and 4(b)).

VEGF and bFGF are the two well-known growth factors secreted by multiple types of cells and function as biochemical cues for wound repair [34, 35]. Both VEGF and bFGF levels were significantly elevated by CoCl_2_ (Figure 3(a)), and CoCl_2_ induced a 2.5-fold increase in VEGF and a 1.6-fold increase in bFGF relative to the nonconditioned MSC sheet which were observed, respectively (Figure 4(a)). The results also revealed that, after decellularization, more amounts of VEGF and bFGF were retained in Cc-ECM than in Nc-ECM (Figures 3(b) and 4(b)).

### 3.4. Enriched ECM from Chemically Hypoxic MSC Sheet Accelerates Wound Healing

**Figure 5:**
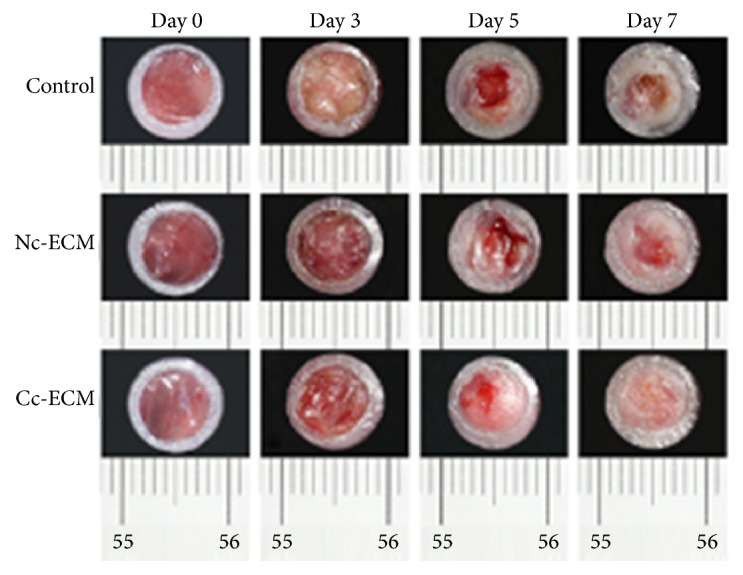
ECMs accelerated wound closure in a mouse model of full-thickness skin defect. Representative images of wound closure during a 7-day in vivo wound healing.

**Figure 6:**
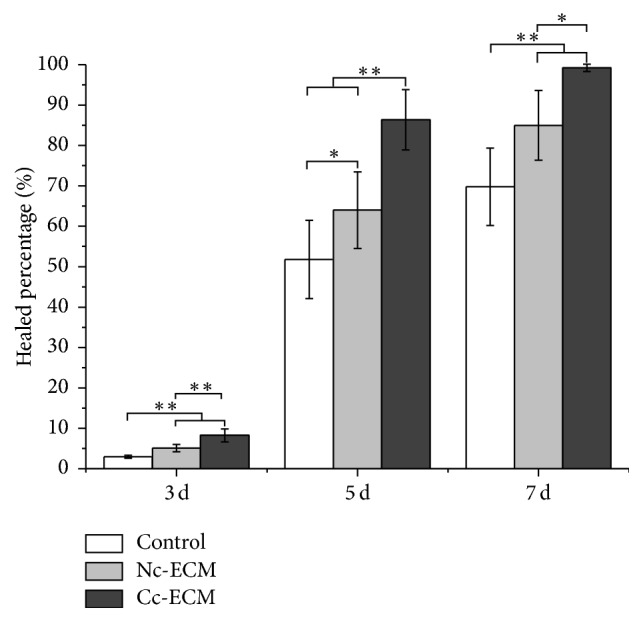
Quantitative analysis of wound closure. Wound closure over time was presented as percentage of healed wound area relative to initial wound area. ^*∗*^*p* < 0.05; ^*∗∗*^*p* < 0.001; *n* = 8.

**Figure 7:**
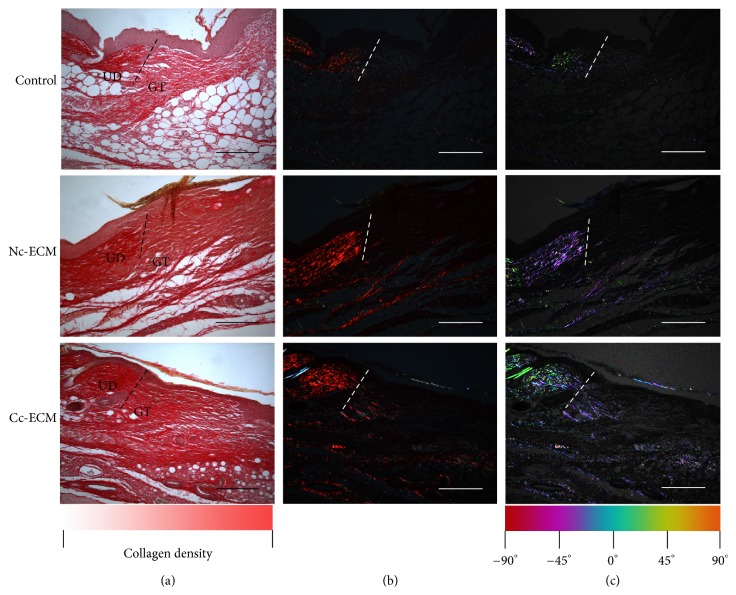
Tissue infiltration and wound edge integration were enhanced with ECMs at day 7 postoperatively. PSR staining of wound edge (UD = uninjured dermis, GT = granulation tissue) (a). Imaging of birefringent collagen through crossed polarization light microscopy. Large orange-red fibers were collagen I and thin blue-green filamentous fibers were collagen III (b). Color maps of fiber orientations were performed using Orientation J software (c). Scale bars represent 200 *μ*m.

Collectively, all the in vitro data showed that conditioning MSC sheet with CoCl_2_ enriched its ECM with both collagens and growth factors. Then a mouse model of full-thickness skin defect was employed to evaluate the in vivo wound healing efficacy of the enriched ECM. Although Nc-ECM did promote wound repair, which were indicated by the higher healed percentages at all time points in its treated wounds comparing with the nontreatment control (Figures 5 and 6), the wound healing property of Cc-ECM was more robust and most of its treated wounds completely healed at day 7 postoperatively, while Nc-ECM-treated wounds healed about 85.0% ± 8.6%, and no-treatment wounds only healed 69.8% ± 9.6% (*p* < 0.05). Moreover, PSR staining showed that the edges of all ECM-treated wounds contained higher collagen content, and thick collagen bundles leading from the uninjured dermis into the granulation tissue were observed (Figures 7(a) and 7(b)). Furthermore, the Cc-ECM-treated wounds exhibited prominent integration of the granulation tissue into the uninjured adjacent dermis. Collagen within the Cc-ECM-treated wound edge was well-oriented, connecting between the surrounding dermis and the granulation tissue, while less such alignment was observed in the Nc-ECM-treated wound edge (Figure 7(c)).

It is noteworthy that no inflammatory signs or visible indication of necrosis was observed in wound tissues during the experimental period, indicating that ECMs derived from xenogeneic MSC sheets induced little immune responses in immunocompetent mice, which was congruent with the previously reported results [36].

### 3.5. Enriched ECM Promotes Early Adipocyte Recruitment and Granulation Tissue Formation

**Figure 8:**
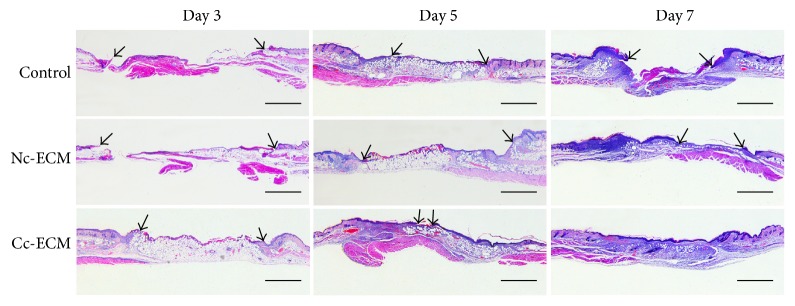
Histological analysis of wound healing. Wound sections on 3, 5, and 7 days after operation were stained with H&E. Black arrows highlighted the epidermal tongue. Scale bars represent 1 mm.

**Figure 9:**
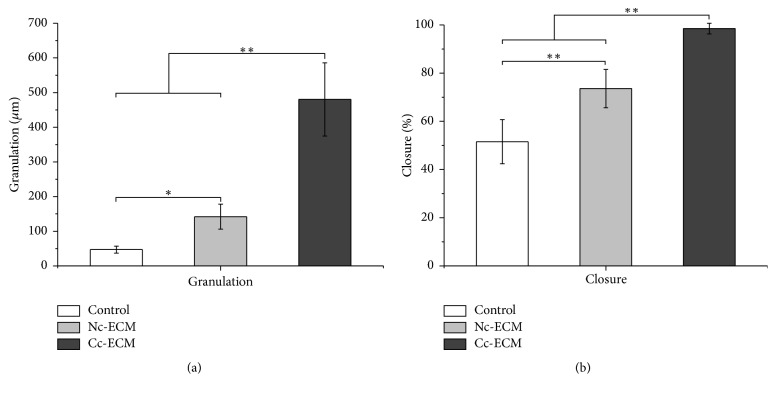
ECMs accelerated granulation tissue formation and reepithelialization. Granulation tissue thickness of the center of wounds at day 7 postoperatively (a). Percentage of closure of the epidermal gap was evaluated at day 7 postoperatively (b). ^*∗*^*p* < 0.05; ^*∗∗*^*p* < 0.001; *n* = 8.

The results of histological analysis demonstrated that adipocytes, which showed empty cytoplasm in H&E-stained sections, filled the wound gap thoroughly on day 3 postoperatively in Cc-ECM-treated mice, partly in the Nc-ECM-treated mice, and scarcely in the nontreated mice (Figure 8). On day 5, adipocytes fully covered the wound bed in the Nc-ECM-treated as well as the nontreated wounds, whereas in the Cc-ECM-treated mice, adipocytes began to disappear and reepithelialization occurred. On day 7, thick and well-structured granulation tissues were observed only in Cc-ECM-treated mice in contrast to little granulation tissues formed in Nc-ECM-treated and nontreated mice (Figures 8 and 9(a)). Although the underlying mechanisms remain unexplored, it seems that early adipocyte recruitment was associated with improved wound repair.

### 3.6. Enriched ECM Promotes Reepithelialization and Angiogenesis

**Figure 10:**
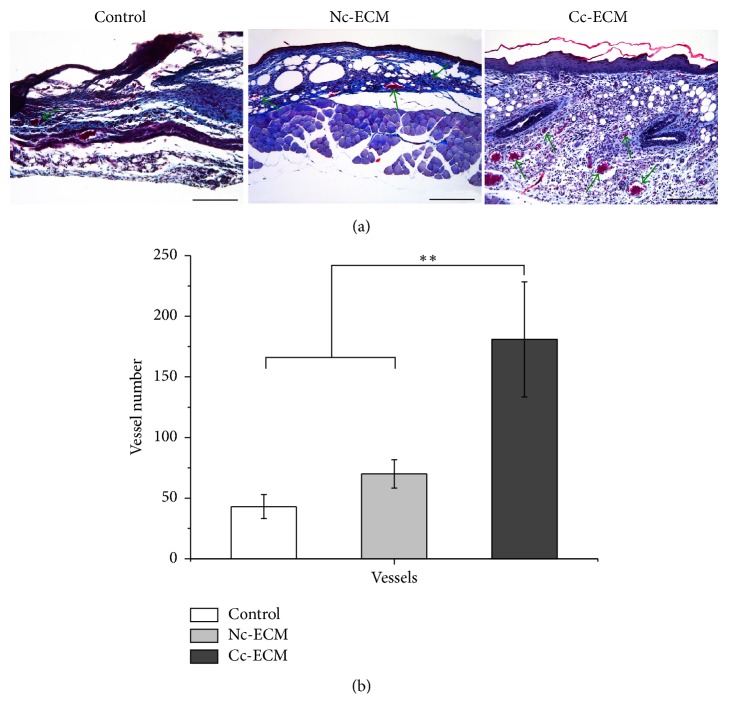
ECMs accelerated angiogenesis. High power field (HPF) (100x) imaging of the center in wound granulation tissues stained with MTC at day 7 postoperatively (a). Scale bars represent 200*μ*m. Number of vessels per HPF at day 7 postoperatively (b). ^*∗∗*^*p* < 0.001; *n* = 8.

When reepithelialization was concerned, we noticed that epidermal cells at the wound edges, which seemed to be originated from adjacent hair follicles, proliferated in all the ECM-treated wounds but not in nontreated wounds at day 3. Moreover, epidermal cells proliferated pronouncedly and began to migrate towards the wound center in Cc-ECM-treated wounds and neoepidermis almost fully covered the wound bed by day 5 (Figure 8), indicating an enhanced reepithelialization, which was consistent with the in vitro analysis of TGF-*β*1 expression. On day 7, although the bilateral epidermal tongues of Nc-ECM-treated wounds migrated significantly (73.6% ± 7.9%) in comparison to that of the nontreated (51.5% ± 9.2%) (*p* < 0.001), large epidermis gap still existed (Figure 9(b)). Furthermore, the total number of vessels counted in Cc-ECM-treated wounds was more than two times of that in Nc-ECM-treated (Figures 10(a) and 10(b)), indicating an augmented angiogenesis which seemed to result from the enhanced retention of VEGF and bFGF in Cc-ECM.

## 4. Discussion

The influence of CoCl_2_-induced chemical hypoxia conditioning of MSC sheets on the wound healing potency of their derived ECMs was investigated and the data revealed that CoCl_2_ conditioning results in ECMs enriched in both collagens and growth factors via a HIF-1*α*-dependent pathway, which exhibit an improved therapeutic efficacy in healing full-thickness skin wounds.

Previous reports showed that MSC treatment by means of intradermal MSC injection, implantation of MSC sheet or MSC-loaded biomaterials, mobilization, and recruitment MSC into the wound beds exerts beneficial effects on cutaneous wound healing through multiple mechanisms [37, 38]. Now it is clear that most of the therapeutic benefits of MSC, such as rapid reepithelialization, pronounced granulation tissue growth, and enhanced angiogenesis, are mainly attributed to its paracrine mediators [14, 15]. In the present study, we showed that MSC sheet-derived acellular ECMs contained abundant collagens and growth factors and significantly promoted wound closure in mice models of full-thickness skin defect, indicating that the ECM portion of MSC secretome possesses active wound healing ability.

It has been reported that hypoxia preconditioning can enhance the tissue repair ability of MSCs [39], and activation of HIF-1*α* has been considered as an efficient strategy to promote wound repair [21]. Moreover, conditioned medium from hypoxic MSCs can also accelerate wound healing [40–42]. So we asked whether exposing MSC sheets to chemical hypoxia could improve the wound healing efficacy of their derived ECMs. Our results showed that CoCl_2_-induced chemical hypoxia was a simple but efficient strategy to activate HIF-1*α*-dependent pathway in MSC sheets and enriched their ECMs with collagens and growth factors by upregulation their expression as well as increasing their preservation during decellularization process. Most of all, the enriched ECMs showed extraordinary in vivo wound healing potency.

ECM-based biologic scaffolds are currently used to fabricate functional organs and tissues to repair, restore, replace, or regenerate damaged tissues or organs. And many robust and effective decellularization protocols have been established to remove all cellular components from a large variety of tissues and organs while maximizing the preservation of their native composition and three-dimensional architectures of the remaining ECMs [43]. In this study, instead of the stringent decellularization procedure for fabricating ADMs from thick dermal tissues [44, 45], a mild procedure was selected to decellularize MSC sheets to minimize the loss of bioactive molecules because they are thinner (thinner than 100 *μ*m) than skin tissues from which ADMs are derived (the thickness of the tissues varies from 300 *μ*m to 3000 *μ*m) [44–49]. Nonetheless, a dramatic loss of both collagens and growth factors in the resulting ECMs was observed. It has been known that the divalent cross-links in collagen are converted into mature and stable trivalent cross-links and thus results in decreased collagen solubility during tissue maturation [50, 51]. Since the Cc-ECM and Nc-ECM were prepared from two-week cultures of MSC sheets, we attributed the loss of collagens and the trapped growth factors to the lack of cross-linking in naive collagen freshly synthesized by MSC. Moreover, lysyl oxidases, which are reported to be upregulated by hypoxia stress and play an important role in initiating collagen cross-linking [52], seemed to contribute to the enhanced retention of both collagen I and collagen III in Cc-ECMs in comparison to Nc-ECMs.

It has been reported that adipocyte lineage cells are activated and intradermal adipocytes repopulate the skin to recruit fibroblast to repair cutaneous wound when the skin integrity is lost [53]. Moreover, defects in adipocyte function by genetic or pharmacological intervention seriously impair wound healing and even result in wound recurrence [53]. In this study, we found that Cc-ECMs which exhibited potent healing properties induced a more rapid adipocyte recruitment compared to Nc-ECMs. And the early adipocyte recruitment is associated with better wound healing outcomes. Recently, it has been revealed that adipocytes are capable of transdifferentiating into myofibroblasts in response to TGF-*β*1 [54, 55], which play key roles in wound repair through ECM deposition and wound contraction [56]. Therefore, it seemed that the TGF-*β*1 trapped in the Cc-ECMs was responsible for their enhanced wound healing abilities. However, the underlying mechanisms of rapid adipocyte recruitment induced by Cc-ECMs are largely unknown.

Both VEGF and bFGF are well-documented growth factors to promote wound repair by promoting keratinocytes proliferation and migration as well as enhancing angiogenesis [17, 35, 57, 58]. In this study, we found that CoCl_2_ conditioning remarkably upregulated VEGF and bFGF, and their abundance in Cc-ECMs was higher than that in Nc-ECMs, which contributed to the improved wound healing efficacy of Cc-ECMs.

## 5. Conclusion

In this study, we showed that CoCl_2_ conditioning activated HIF-1*α* and significantly increased the expression of both collagens and growth factors in MSC sheets and hence resulted in enriched ECMs with potent in vivo wound healing properties. Although future research should be carried out to optimize decellularization process to retain more growth factors and collagens in the enriched ECMs, it is hoped that the bench-to-bedside translation of the present work will lead to a readily available MSC product that can be clinically used to manage skin injuries.
